# Metformin alters H2A.Z dynamics and regulates androgen dependent prostate cancer progression

**DOI:** 10.18632/oncotarget.26457

**Published:** 2018-12-11

**Authors:** Monica Tyagi, Manjinder S. Cheema, Deanna Dryhurst, Christopher H. Eskiw, Juan Ausió

**Affiliations:** ^1^ Department of Biochemistry and Microbiology, University of Victoria, Victoria, BC, Canada; ^2^ ImmunoPrecise Antibodies Ltd., Victoria, BC, Canada; ^3^ Department of Food and Bioproduct Sciences, University of Saskatchewan, Saskatoon, SK, Canada

**Keywords:** metformin, prostate cancer, H2AZ, androgen receptor

## Abstract

Epigenetic mechanisms involved in prostate cancer include hypermethylation of tumor suppressor genes, general hypomethylation of the genome, and alterations in histone posttranslational modifications (PTMs). In addition, over expression of the histone variant H2A.Z as well as deregulated expression of Polycomb group proteins including EZH2 have been well-documented. Recent evidence supports a role for metformin in prostate cancer (PCa) treatment. However, the mechanism of action of metformin in PCa is poorly understood. We provide data showing that metformin epigenetically targets PCa by altering the levels and gene binding dynamics of histone variant H2A.Z. Moreover, we show that the increase in H2A.Z upon metformin treatment occurs preferentially due to H2A.Z.1 isoform. Chromatin immunoprecipitation (ChIP)-RT PCR analysis indicates that metformin treatment results in an increased H2A.Z occupancy on the androgen receptor (AR) and AR-regulated genes that is more prominent in the androgen dependent AR positive LNCaP cells. Repression of H2A.Z.1 gene by siRNA–mediated knock down identified this H2A.Z isoform to be responsible. Based on preliminary data with an EZH2-specific inhibitor, we suggest that the effects of metformin on the early stages of PCa may involve both EZH2 and H2A.Z through the alteration of different molecular pathways.

## INTRODUCTION

Epidemiological studies have reported that prostate cancer (PCa) remains the most commonly diagnosed, non-cutaneous cancer in males [1] and is the second leading cause of death after lung cancer in western populations of men. The first line of treatment for PCa is androgen deprivation therapy (ADT); however, with the advancement of the disease, almost all patients eventually progress towards castration resistant prostate cancer (CRPC) [2]. It is noteworthy, that in the majority of cases, disease progression to CRPC relies on a functional androgen receptor (AR) signaling cascade [3].

Over a decade of research demonstrates the positive influence of biguanide drugs on the prevention and treatment of a wide range of cancer types, including PCa. Metformin (1,1-dimethylbiguanide hydrochloride) is the most commonly used insulin sensitizer that belongs to the biguanide oral hypoglycemic family [4, 5]. Clinical records mark metformin as a safe drug with limited toxicity, wherein 90% of metformin is eliminated through the renal system. A cohort study of 8,000 type 2 diabetes patients demonstrated that individuals not taking metformin showed 4.3% higher diagnosed cancer cases compared to metformin users [6]. Based on a population study, Margel *et al.* demonstrated an association between metformin usage and improvement in survival among older men with diabetes and PCa [7]. One of the measurable effects of metformin involves the activation of the tumor suppressor gene liver kinase B1 (LKB1), a known regulator of AMP-activated protein kinase (AMPK) [8]. The activation of LKB1 and AMPK ultimately lead to the inhibition of the mammalian target of rapamycin (mTOR) pathway [9] that mediates anti-tumor activity of metformin. Recent reports have established c-MYC, enhancer of zeste homolg 2 (EZH2) and androgen receptor (AR) as targets of metformin [10–12].

Androgen receptor (AR) is critical to the molecular etiology of prostate cancer progression [13]. AR belongs to the nuclear receptor family that when inactive are sequestered in the cytoplasm. Upon androgen stimulation, AR undergoes conformational changes leading to its homodimerization, translocation to the nucleus, and binding to DNA androgen response elements (ARE), thereby regulating downstream gene expression [14]. Additionally, activated ARs recruit co-activator proteins such as SNF2-related CBP activator protein (SCRAP), a chromatin remodeling complex that is responsible for incorporation of H2A.Z-H2B heterodimers into chromatin [15, 16].

H2A.Z is a histone variant found in association with gene regulatory regions including promoters and enhancers [17, 18], and regulates cell proliferation [19]. Our group previously reported the identification of two H2A.Z isoforms: H2A.Z.1 and H2A.Z.2 [20] and described the association of H2A.Z.1 with androgen receptor dependent prostate cancer progression [21]. MYC is a transcription factor involved in cell cycle regulation which is dysregulated in many cancers [20] and the H2A.Z.1 gene promoter contains several MYC transcription factor binding sites [22] implicating c-MYC binding as a possible mechanism to facilitate increased cell levels of H2A.Z.1 deposition.

EZH2 is a master epigenetic transcriptional regulator of many cancers [23] including PCa [24, 25] and is an integral component of the Polycomb Repressive Complex 2 (PRC-2). As with histone H2A.Z [26], EZH2 gene expression is stimulated by MYC [22]. EZH2 catalyzes the addition of methyl groups to histone H3 at lysine 27, a histone PTM usually associated with chromatin condensation [27] and gene repression. At advanced stages of prostate cancer progression, EZH2 can acquire an oncogenic function which is independent of its polycomb-associated transcriptional repressor activity [23] as in CRPC cells [25]. In such instances, EZH2 works instead as a co-activator in conjunction with transcription factors such as AR [25].

The involvement of H2AZ and EZH2 in prostate cancer and the reported down regulation of AR gene [11] by metformin prompted us to further analyze the role of this drug in these two important epigenetic components. We demonstrate that androgen dependent prostate cancer lymph node carcinoma of the prostate (LNCaP) cells show an increase in H2A.Z both at the protein and transcript levels upon treatment with metformin. In addition, ChIP-qPCR showed an increased occupancy of H2A.Z at several regions of the *AR* and prostate specific antigen (*PSA*) genes. This enhanced interaction of H2A.Z with *AR* resulted in a dysregulation of its expression and of AR cellular levels, which were lowered, as confirmed by H2A.Z knockdown experiments (Figure 5). These results reveal a novel role of metformin at the epigenetic level and open up important questions as to the detailed molecular mechanisms involved. This information will provide an essential foundation to establish and understand the mechanisms of how metformin potentiates the effects of specific gene targets, which can then be used to explore its potential use for an effective approach in diagnosis and treatment of prostate cancer.

## RESULTS

### Effects of metformin on cell clonogenicity

**Figure 1:**
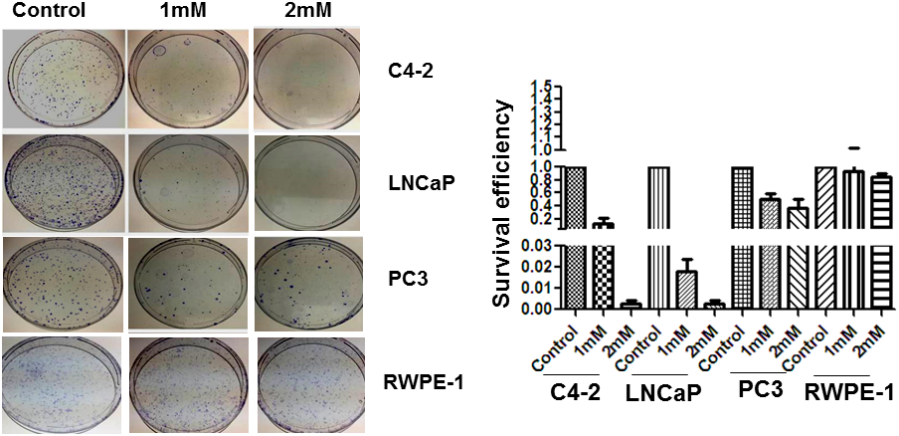
Colony formation assay Colony formation of C4-2, LNCaP, PC3 and RWPE-1 cells after 1mM and 2mM of metformin treatment. Cells were seeded into 100 mm plates at 500-2000 cells per plate and were then treated with different doses of metformin for 24 hr. Cell survival was determined by colony formation assay. The data is a representation of three independent experiments that were quantified using image J software.

Colony formation assays were performed in plates of LNCaP (AR positive, androgen dependent), RWPE-1 (non-cancerous), PC-3 (AR negative) and C4-2 (AR positive, androgen independent) in response to metformin treatment (1 mM and 2 mM). Clonogenic assays revealed that RWPE-1 and PC-3 retained a high level of clonogenicity after 1 mM and 2 mM metformin treatment, whereas LNCaP and C4-2 cells had greatly reduced clonogenicity under the same conditions (Figure 1). When seeded at 2,000 cells/plate and treated with 1mM metformin, the survival efficiency of LNCaP cells was 10-fold less than C4-2 and PC-3; however, at the higher dose of 2 mM, LNCaP and C4-2 cells showed significant decrease of 80-90-fold in their survival efficiency compared to the untreated controls (Figure 1). Moreover, the effect of metformin treatment was minimal on RWPE-1 and PC-3 prostate cancer cells indicating that lack of AR protein in normal and negative AR cells negates the effects of metformin on cell death. These results indicate that the metformin-mediated inhibitory effect on clonogenicity is high in LNCaP and C4-2 with LNCaP cells being the most sensitive at 1 mM concentration when compared to the three other three cell lines.

### Metformin alters the levels of H2A.Z, EZH2 and H3K27me3

**Figure 2:**
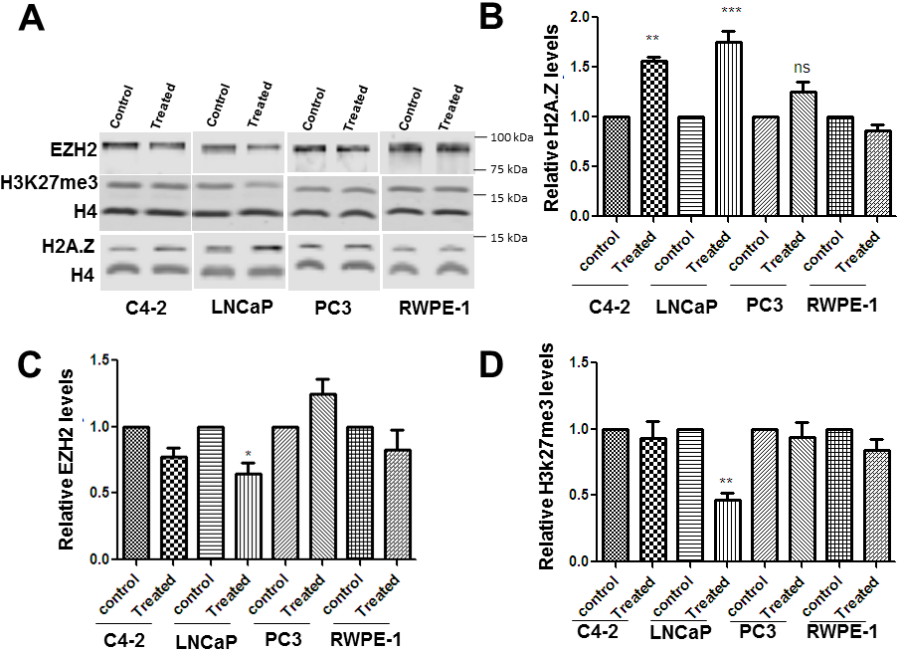
Metformin exposure alters the levels of H2A. Z, EZH2 and H3K27me3 levels in PCa cells Four prostate cell lines (C4-2, LNCap, Pc-3 and RWPE-1) were treated with 2 mM metformin for 24hrs. **(A)** Representative western blot analyses for H2A.Z, EZH2, H4 and H3K27me3 obtained from cell protein lysates of metformin treated and untreated (control) cells. **(B, C and D)** Bar-plot representation of the western blot data obtained from four independent experiments normalized using H4. Student’s t- tests were used to calculate significance; P value, ^*^ *P* < 0.05, ^**^P < 0.01, ^***^P < 0.001.

The effect of metformin treatment on the levels of H2A.Z, EZH2 and H3K27me3 in prostate cancer cells (LNCaP, C4-2, and PC-3) and in normal (RWPE-1) cells was examined by western blotting. The data (Figure 2A-2B) shows a significant increase in the levels of H2A.Z upon metformin treatment in LNCaP and C4-2 cell lines. In PC-3 and RWPE-1 cells there was not much change observed in the levels of H2A.Z. By contrast, EZH2 and H3K27me3 levels (Figure 2C and Figure 2D) were decreased upon metformin treatment in LNCaP cells with no significant changes observed in the other three cell types. Based on these results, we suggest that the metformin-induced alteration in levels of these three proteins preferentially affects the AR-positive, androgen dependent LNCaP cells.

### Metformin treatment leads to a differential expression of H2A.Z isoforms

**Figure 3:**
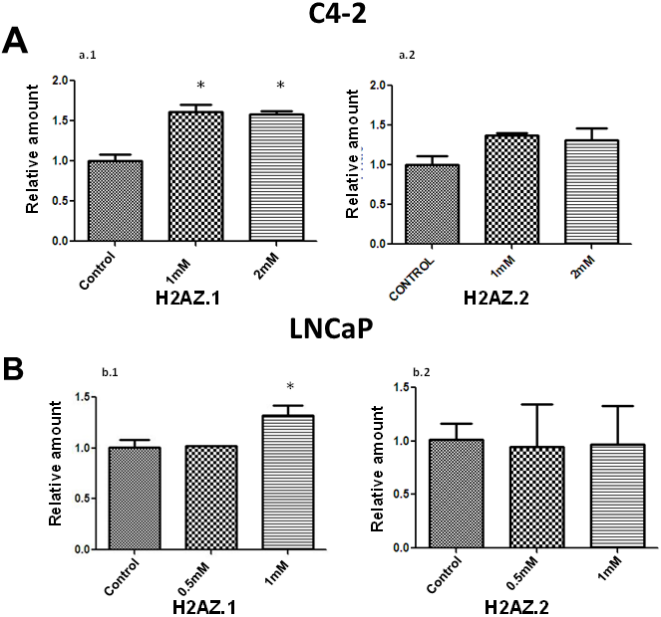
Metformin treatment preferentially increases the expression of H2AZ 1.RT-PCR analyses of the expression of H2A.Z.1 and H2A.Z.2 upon metformin treatment of C4-2 **(A)** and LNCaP **(B)** PCa cells. The data was obtained from three independent experiments. GAPDH was used as a normalizer for these analyses. Student’s t- test was used for statistical analysis and to calculate the significance; ^*^ *P* < 0.05.

The H2A.Z variant has two isoforms; H2A.Z.1 and H2A.Z.2 [20, 31] and remarkably, both have their own functional importance [32, 33]. The increase observed in H2A.Z levels upon treatment with metformin in LNCaP and C4-2 cells prompted us to determine whether the expression of both isoforms was altered. RT-PCR performed on control and metformin-treated LNCaP and C4-2 cells (Figure 3A and 3B) shows an increase in the expression of H2A.Z.1 upon metformin treatment. However, no significant alteration was observed in the levels of H2A.Z.2 transcripts. The data strongly indicates that the alteration of H2A.Z.1 isoform expression is responsible for the overall increase in the level of H2A.Z which is observed in these two cell lines as a result of metformin treatment.

### Metformin affects H2A.Z gene occupancy

**Figure 4:**
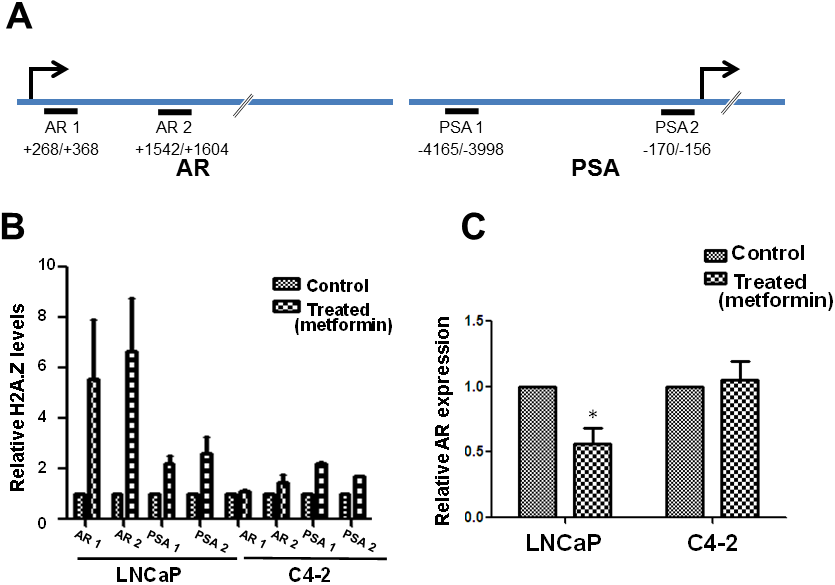
Metformin treatment alters H2A. Z gene occupancy and AR expression **(A)** Schematic representation of the AR and PSA genes indicating the regions used for the reparation of the primers used in the analyses. **(B)** H2A.Z occupancy at different regions (AR 1, AR 2, PSA 1 and PSA 2) of the AR and PSA genes in LNCaP and C4-2 cells as determined by ChIP-RT-PCR analyses. Histone H3 contents was used as a normalizer for occupancy. **(C)** Graphical representation of the levels of androgen receptor expression determined by q-RT-PCR analyses of metformin treated and untreated (control) C4-2 and LNCaP cells using GAPDH as a normalizer. The data is a representation of three independent experiments. Student’s t- tests were used to calculate significance; P value, ^*^ *P* < 0.05, ^**^P < 0.01, ^***^P < 0.001.

The increase of H2A.Z observed in LNCaP (androgen dependent) and C4-2 (androgen independent) PCa cells led us to investigate the effect(s) of metformin on the total H2A.Z gene occupancy in these cell types. The AR-responsive *AR* (androgen receptor) and *PSA* genes were chosen because of their direct and indirect involvement in PCa progression respectively. AR regulates transcription of these genes by binding to their androgen response elements (AREs), as is exemplified by *PSA* [34, 35]. Moreover, information is already available for the H2A.Z occupancy of *PSA* in the absence of metformin treatment [21]. Select regions within these two genes were chosen for ChIP-qPCR analysis (Figure 4A). The *AR* gene is 90 kb long and encodes a 110 kDa AR protein that has three major functional domains: The N-terminal domain, DNA-binding domain, and androgen-binding domain. It contains six exons of which exon-1 codes for the entire N-terminal domain that encompasses the activation functiona1 element which is crucial for transcriptional activation [36] and for the regulation of androgen dependent prostate cancer development [37]. Hence, we chose to analyze H2A.Z localization on different regions (AR1: +268/+236, AR2: +1542/+1604) of exon-1 in this gene. For the *PSA* gene the already established H2A.Z-containing nucleosome regions at an ARE within the enhancer (PSA1: -4165/-3998) and within the proximal promoter (PSA 2: -170/-156), corresponding respectively to PSA B and PSA D regions previously identified [21] were used (see Figure 4A and Table 1 for the primers utilized).

**Figure 5:**
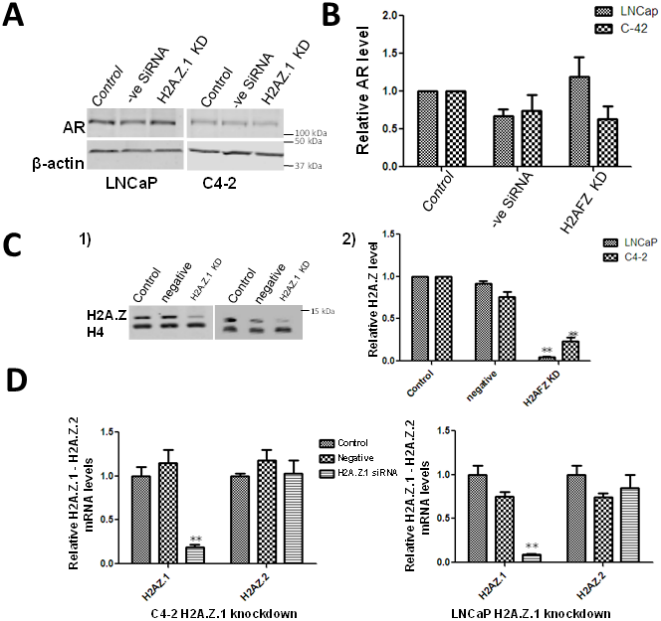
Effect of H2A. Z.1 (H2AFZ) knock down on the levels of AR **(A)** Western blot analyses of siRNA-mediated knockdown of H2A.Z.1 (H2AFZ) in LNCaP and C4-2 cells. The change in levels of AR and H2A.Z were normalized using β-actin. **(B)** The data from 3 independent experiments is graphically represented using GraphPad Prism version 5 software. Student’s t- tests were used to calculate significance; P value, ^*^ *P* < 0.05, ^**^P < 0.01, ^***^P < 0.001. Starting cells: (control), cells transfected with a scrambled non-targeted siRNA: (negative siRNA) and H2A.Z.1 siRNA (H2A.Z.1 KD). **(C)** Western blot analysisof protein lysates obtained from starting LNCaP/C4-2 (control), cells transfected with a scrambled siRNA (negative) and cells transfected with H2A.Z.1 siRNA. **(C-1)** Image of a representative blot. **(C-2)** Quantitative analysis using histone H4 as a normalizer. The figure shows the average of three independent experiments. Student’s t- tests were used to calculate significance; P value, ^*^ *P* < 0.05, ^**^P < 0.01, ^***^P < 0.001. **(D)** qRT-PCR analysis of H2A.Z.1 knock down efficiency and specificity in C4-2 and LNCaP cells.

**Table 1:** 

Gene name	Primer sequence
**Primers for reverse transcriptase PCR**	
*H2AZ.1*	F: TCCGGAAAGGCCAAGACAA R: TGTCGATGAATACGGCCCAC
*H2AZ.2*	F: CCGCATCCACAGACACTTGA R: GGTACTCCAGAATCGCAGCA
*GAPDH*	F: AAGGTCATCCCTGAGCTGAACGGG R: CCAGGAAATGAGCTTGACAAAGTG
*PP1*	F:GGTGACTTCACACGCCATAA R: GTCTTGGCAGTGCAGATGAA
*AR*	F: AGCAGGGTGAGGATGGTTCT R: AGGTTGCTGTTCCTCATCCA
**Primers for ChIP qPCR**	
*PSA 1*	F: ACTGGGACAACTTGCAAACC R: TCTCAGATCCAGGCTTGCTT
*PSA 2*	F: CTGCCTTTGTCCCCTAGATG R: AAACCTTCATTCCCCAGGAC
*AR 1*	F: AGCAGGGTGAGGATGGTTCT R: AGGTTGCTGTTCCTCATCCA
*AR 2*	F: CCCAGTCCCACTTGTGTCAA R: AAACTTACCGCATGTCCCCG

The ChIP-qPCR data (Figure 4B) show that in metformin-treated LNCaP cells, H2A.Z occupancy increased 4-5 fold upon metformin treatment compared to a slight increase in C4-2 cells on the two selected *AR* regions. A similar enrichment of H2A.Z was observed, albeit to a much lower extent, at the *PSA* (PSA1 and PSA2) regions of both LNCaP and C4-2 cell lines. Based on this, we suggest that there is significant enrichment of H2A.Z at different regions of androgen regulated genes after metformin treatment, which is more prevalent in androgen dependent LNCaP cells.

The effect of metformin on *AR* expression under the conditions used for the ChIP-qPCR approach was analyzed next and the data showed a decrease in its expression (Figure 4C). These results are in agreement with an earlier report [11].

### H2AZ.1 knockdown affects AR expression

Our results show a significant impact of metformin treatment on the deposition of H2A.Z at the AR gene. This treatment also results in an overall increase in H2A.Z (Figure 2B) which is due to H2A.Z.1 isoform (Figure 3). Therefore, we analyze if these changes could affect the AR expression by performing H2A.Z.1 knockdown. The efficiency of the knockdown was confirmed both at the protein and RNA levels (Figure 5). The western blot data of the H2AZ.1 knockdown carried out in LNCaP cells shows an increase in the levels of AR in these cells when compared to the untransfected cell control or to a vehicle control of cells transfected with a non-specific siRNA (Figure 5). No significant change was observed in AR expression when the same approach was carried out in C4-2 cells. This led us to suggest that the decrease in H2A.Z.1 levels occurs concomitantly with increased expression of the AR gene in metformin-treated androgen dependent LNCaP cells.

### Inhibition of EZH2 modulates the metformin-mediated H2A.Z increase

**Figure 6:**
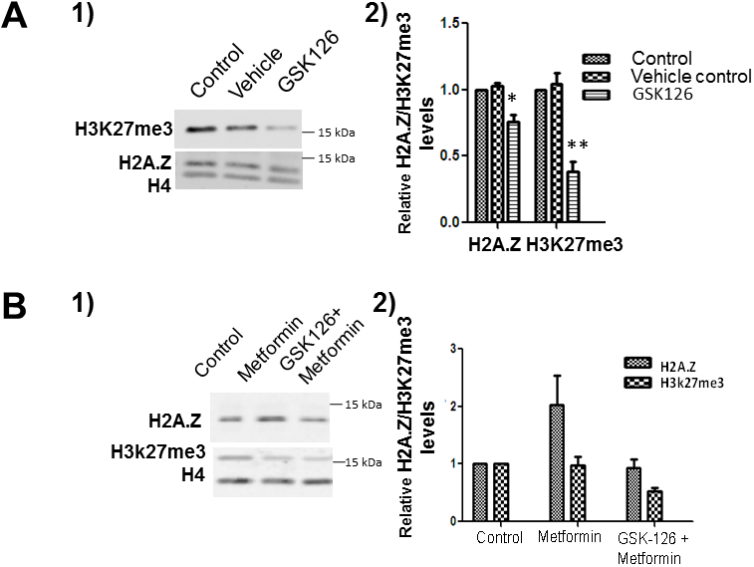
EZH2 inhibition decreases the levels of H2A. Z **(A)** Western blot analyses of control, vehicle and GSK126 treated LNCaP cells immunoblotted using EZH2, H3K27me3 and H2A.Z antibodies. **(A-1)** Representative image. **(A-2)** Graphical representation of the western blot data shown in (A-1). The data is a representation of three independent experiments normalized using H4. **(B)** LNCaP cells were exposed to GSK126 and subsequently treated with metformin. Protein lysates obtained from control, metformin and a combination of GSK126 and metformin treated cells were analysed by western blot using H3K27me3 and H2A.Z antibodies. **(B-1)** Representative western blot image. **(B-2)** Graphical representation of the western blot data from 3 independent experiments. Student’s t- tests were used to calculate significance; P value, ^*^ *P* < 0.05, ^**^P < 0.01, ^***^P < 0.001. H4 was used as a normalizer. Control: Starting LNCaP/C4-2 cells; Vehicle: DMSO; GSK 126: GSK 126 treated cells.

Since both H2A.Z and EZH2 are chromatin-associated transcriptional regulators whose expression is affected by metformin (Figure 2), we tested if there was a functional link between the two. We performed an assay using GSK126, a well-known inhibitor of EZH2. LNCaP cells were treated with GSK126 and levels of H2A.Z and H3K27me3 were monitored using western blots (Figure 6A-1). The efficiency of the inhibition was confirmed by the decrease in H3K27me3 on both GSK126 treated cell lines.

To further investigate the influence of EZH2 inhibition on metformin, western blot analysis was carried out on protein extracts isolated from LNCaP cells following 24 hour GSK126 treatment, and subsequent 24 hour metformin treatment. As seen in Figure (6B-1)-(6B-2) EZH2 inhibition resulted in a loss of H2A.Z induction in the presence of metformin, when compared to untreated control and in response to metformin alone. This data is surprising and we suggest an involvement of EZH2 in the regulation of H2A.Z levels in androgen dependent PCa cells. However, this represents very preliminary data and further studies will be needed in order to validate of the association of these two proteins.

## DISCUSSION

### Metformin decreases the viability of LNCaP and C4-2 PCa cell lines

In agreement with previous reports [12, 29, 38–43], treatment of AR positive cell lines LNCaP and C4-2 with metformin restricted their growth. We found the androgen dependent LNCap cells to be more sensitive than the androgen independent C4-2 cells in the 1-2 mM range of concentration (Figure 1). The more aggressive AR negative PC3 cell type exhibited only a slight decrease in clonogenicity, whereas in RWPE-1 cells metformin treatment had minimal effect. Hence, the metformin inhibitory effect observed with these cell lines, which presumably involves the stimulation of cell cycle arrest and apoptosis, is specific to cancerous cells with a functional AR signaling axis [12].

### Metformin alters the levels of histone variant H2A.Z.1 in prostate cancer cells

Metformin has been reported to have a regulatory role in controlling the progression of PCa from androgen dependence to androgen independence by targeting the AR [11], however, the intricate molecular details behind this observation remain to be determined. Metformin has been reported to activate the AMPK (AMP-activated protein kinase) pathway and modulate glucose and fat synthesis that are essential for tumor growth [44, 45]. Nevertheless, how this trickles down to chromatin to regulate gene expression is not well understood. We took advantage of the previous knowledge generated by our group which indicated that alterations in the levels of H2A.Z participate in PCa progression [21, 26], to analyze whether the levels of this histone variant could also be affected by metformin. Histone variant H2A.Z is an important chromatin transcriptional regulator which can epigenetically affect gene expression [46]. Upon exposure of AR positive PCa cells (LNCaP and C4-2) to metformin, we observed an increase in H2A.Z both at protein and RNA levels (Figures 2-3). Importantly, such increase can be attributed to the H2A.Z.1 isoform of H2A.Z (Figure 3 and Figure 5) which has been shown to be particularly involved in PCa [21].

The effect of metformin on the levels of H2A.Z within the context of PCa is not surprising as histone variants have been shown to play a very important role in the progression of many types of cancer. For instance, the levels of macroH2A.1.1 and macroH2A.2 can be used as predictors for the risk of lung cancer recurrence [47] and increased expression of macroH2A1.1 correlates with poor prognosis of triple negative breast cancer. Also, histone H3.3 promotes lung cancer cell migration [48]. Histone H2A.Z and its isoforms H2AZ.1 and H2AZ.2 alterations have been shown to be involved in melanoma [49], breast [50] and prostate cancers [21]. However, what remains to be elucidated is how metformin can alter the levels of H2A.Z and how this can impacts PCa biology.

### A potential molecular mechanism for the metformin-mediated increase in H2A.Z

**Figure 7:**
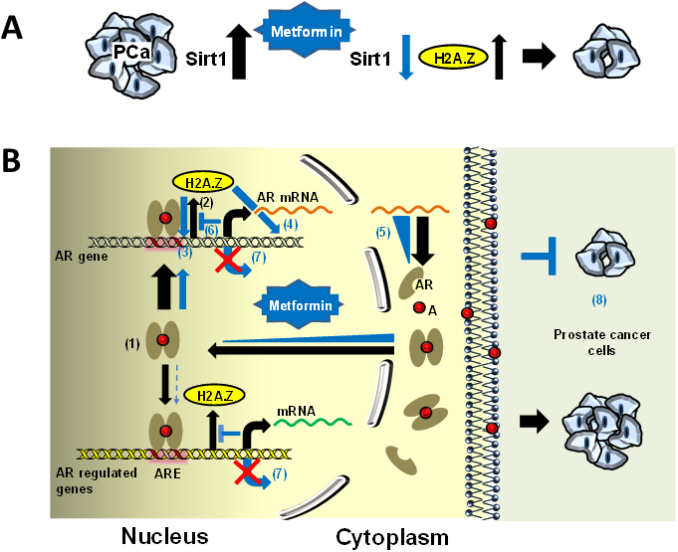
Schematic representation of the potential molecular mechanism behind metformin mediated increase in H2A. Z and dynamics in PCa **(A)** SIRT1 is overexpressed in PCa [52] (black thick arrow). Metformin treatment down-regulates the Sirt1/Pgc-1α/Nrf2 [54] and hence the levels of SIRT1 (blue arrow) which results in an increase in H2A.Z (black arrow)[55] and a restriction in tumor growth. **(B)** Androgen receptor (AR) is overexpressed in prostate cancer cells and up-regulates the expression of AR-dependent genes (1). During the process histone variant H2A.Z is released from promoters and enhancer regions (2) [21]. Upon metformin treatment, the occupancy of H2A.Z observed at the promoter regions and in different regions of these genes increases (3-4) and results in a reduction of their expression (5). The increase in H2A.Z particularly observed at promoter regions (3) is surprising and we suggest that this is the result of an antagonistic mechanism with androgen (6) which represses the expression of these genes (7). Hence, H2A.Z plays an important role in restricting tumor growth (8). The black arrows indicate the pathway involved in AR response and the blue arrows and triangles denote the alterations resulting from metformin treatment. Abbreviations: A, androgen; ARE, androgen responsive element.

Sirtuin 1 (Sirt1) is an NAD+ dependent histone deacetylase which has been shown to play a very important role in the initiation and progression of many cancers [51] including PCa [52, 53]. This is mediated through the critical role it plays in oxidative stress and cellular redox balance and metformin has been shown to down-regulate the Sirt1/Pgc-1α/Nrf2 (sirtuin 1/peroxisome proliferator-activated receptor γ co-activator 1α/nuclear erythroid related factor 2) pathway. This in turn leads to the increased susceptibility of p53 positive cancer cells (such as LNCaP and C4-2) to oxidative stress and therapeutic agents [54]. Interestingly, SIRT1 and H2A.Z deregulation in PCa have been shown to be reciprocally related [55]. Hence, the overall increase in H2A.Z observed by us upon metformin treatment of LNCaP and C4-2 would be in agreement (Figure 7A) and could provide an explanation for our observations (Figure 7A).

Regardless of the cause responsible for the metformin-mediated H2A.Z increase observed in LNCaP and C4-2 cells, an important remaining question has to do with the potential molecular mechanism(s) by which such increase mediates the observed metformin effects on LNCaP and C4-2 cell growth suppression (Figure 1 and Figure 7A).

### Metformin alters the H2A.Z nucleosome occupancy and expression of AR regulated genes

Histone variants affect the dynamics with which histones interact with gene regulatory regions (promoters, enhancers) and alter gene expression [21, 49, 56]. For instance the position occupied by H2A.Z at the +1 or -2 nucleosome flanking the transcription starting site (TSS) of gene promoters can respectively correlate with their repressive or activating nature [57]. Hence, alterations in the genome occupancy of histone variants can affect the functionality of the targeted chromatin domains. Therefore, the overall increase observed in the levels of H2A.Z in LNCaP and C4-2 cells upon metformin treatment may have important functional implications in terms of gene occupancy and dynamics as will be described next.

We questioned whether the metformin-mediated increase in the levels of H2A.Z also resulted in the larger presence of this histone variant in the nucleosomes of the AR targeted genes. To address this, ChIP-qPCR was performed and occupancy of H2A.Z at androgen receptor and PSA gene promoters, two key players in PCa progression, were analyzed (Figure 4B). It had previously been shown that, in the case of the PSA gene, histone H2A.Z is present at the enhancer and promoter regions of the gene where the AREs are located. Moreover, it has now been shown that the levels of H2A.Z decrease dramatically upon androgen induction [21] (Figure 7B: (2)). Coupling between loss of H2A.Z at gene regulatory sites and transcriptional activation has been well documented [58, 59]. Hence, it is possible that the increase in H2A.Z occupancy observed in different regions of the AR responsive genes, including *AR* itself (Figure 7B: (3)-(4)), results in the reduction of their expression as a consequence of their repressed chromatin architecture (Figure 7B: (5)-(7)). In this regard the lack of expression observed in the case of the AR gene in LNCaP cells would not be that surprising (Figure 4C).

In addition to all this, the metformin-mediated increase in H2A.Z could also be accounted for by the well-known effect of metformin in the induction of the DNA damage repair (DDR) pathway [60]; a process in which H2A.Z has also been shown to be involved [61].

### Further epigenetic complexity of metformin treatment

Figures 2C-2D show that in addition to increasing the levels of H2A.Z (Figure 2B), metformin also decreases the levels of EZH2 and consequently decreases H3K27me3 particularly in LNCaP cells. Hence, as will be briefly discussed next, the epigenetic changes imparted by metformin are more complex and dynamic in regulating the chromatin landscape than originally anticipated. While the increase in the levels of H2A.Z appears to have a repressive activity on AR responsive genes as described above, the decrease observed in both EZH2 and H3K27me3 in LNCaP cells upon metformin treatment would indicate an opposite effect, which is most likely affecting a different set of genes. In fact, EZH2 may operate through a completely different mechanism(s) and the effect of metformin on this enzymatic transcriptional regulator may involve the conventional metabolic AMPK pathway [62]. In support of this, it has been shown that EZH2 phosphorylation by AMPK acts as a suppressor of polycomb repressive methyltransferase activity and oncogenic function [63].

Our preliminary experiments (Figure 6) using GSK126, a well-known inhibitor of EZH2 methyltransferase activity [64], hint at the possibility that the effects of metformin observed on LNCaP cells might involve the synergistic action of the transcriptional enzymatic (EZH2) and structural (H2A.Z) regulators.

### Concluding remarks

As mentioned earlier, the relevance of histone variant H2A.Z to PCa has been quite extensively studied [21, 56, 58, 59, 65, 66]. The work described in this paper is the first report establishing role of a histone variant mediating the effects of metformin in PCa. The androgen responsive AR positive LNCaP cells appear to be predominantly targeted. This hints at a prevalent impact of the drug in the early stages of PCa progression before becoming androgen non-responsive on par with its more modest clinical relevance with metastatic castration-resistant activity [67]. In this regard it would be of interest in the future to analyze the combined effects of this drug with other drugs such as enzalutamide [68].

## MATERIALS AND METHODS

### Cell culture and reagents

The human prostate cancer cells: PC3, LNCaP, RWPE-1 and C4-2 cells were kindly provided by Dr. Rennie’s lab at the Vancouver Prostate Centre. LNCaP and C4-2 cells were maintained in Roswell Park Memorial Institute (RPMI) 1640 medium (Gibco), PC3 cells were maintained in Dulbecco’s modified essential medium (DMEM) (Gibco) supplemented with 10% Bovine Growth Serum (BGS) (VWR), 1% penicillin/streptomycin (Sigma-Aldrich) and 1X Gibco GlutaMAX (ThermoFisher Scientific). RWPE-1 cells were maintained in Keratinocyte Serum Free Medium (K-SFM) supplemented with bovine pituitary extract (BPE) and human recombinant epidermal growth factor (EGF) (Gibco) with 10% BGS and 1% penicillin/streptomycin. Cells were cultured at 37°C in a humidified atmosphere of 5% CO_2_.

Metformin (Sigma-Aldrich) was dissolved in sterile water at a 100 mM concentration stock. Based on the cell type sensitivity in colony formation assay, LNCaP cells were treated at a concentration of 0.5 mM and 1 mM, and C4-2 cells were treated at a concentration of 1 mM and 2 mM for 24 hrs. Cells were treated at the aforementioned concentrations [28, 29] based on the cell type sensitivities to each dosage for 24 hrs. GSK 126 (Cayman chemicals) was dissolved in DMSO at a 1 mM concentration stock. The cells were treated with a drug concentration of 2 μM or vehicle control (DMSO) for 48 hrs. The similar concentrations of metformin and GSK126 were used in combination as well as for studying the effect of EZH2 inhibition on H2A.Z levels post metformin treatment.

### Colony formation assay

The confluent cells were trypsinized and plated at densities ranging from 500 to 2000 cells per 10 cm petri dish, based on the doubling time of each cell line [30]. Cells were allowed to adhere and undergo one cycle of cell division before metformin treatment (1-2 mM) was done for 24 hrs. The cell culture media was changed every 3-4 days. Colonies developed after 10-15 days, then were fixed by acetic acid: methanol solution (1:7) for 5 minutes at room temperature. For visualization, the colonies were stained with 0.5% crystal violet for 2 hours at room temperature, rinsed, air-dried and quantified using ImageJ software. Each experimental replicate was internally controlled using untreated cells. The relative plating efficiencies were expressed as percentages relative to the plating efficiency of untreated cells.

### Immunoblotting

Protein lysates were prepared by mixing a volume of cell suspension with an equal volume of 2X Laemmli buffer (2% SDS, 10% glycerol, 0.002% bromophenol blue and 62.5mMTris-HCl pH 6.8). Protein content was then determined by the Lowry method (Bio-Rad Hercules). Immediately after, 3% β-mercaptoethanol was added; three to six μg of protein lysate was separated by sodium dodecyl sulfate polyacrylamide gel electrophoresis (SDS-PAGE), and transferred onto nitrocellulose membranes (GE Healthcare). Nonspecific binding was blocked by incubation in 5% skim milk in phosphate buffered-saline pH 7.2 with 0.1% Tween 20. Membranes were incubated with specific antibodies, either overnight at 4°C or for 1 hour at room temperature. The membranes were probed with different primary antibodies: H2A.Z 1:2,000 (Abcam, cat#ab4174), EZH2 1:2000 (Cell signaling cat#D2c9), H3K27me3 1:3,000 (Milliporecat#07-449), AR 1:500 (Santacruz,cat#Sc-7305), β-actin 1:20,000 (A2228, Sigma) and H4 1:20,000 (rabbit serum produced in-house), followed by incubation with a secondary antibody conjugated to fluorescent dye, IRDye 800 Anti-rabbit IgG 1:10,000 (Rockland Antibodies, cat#611-132-122). Immunoblots were visualized using Li-Cor Odyssey (LI-COR Biosciences). Images were analyzed using Li-Cor Image Studio version 5.2 software.

### Real time polymerase chain reaction (RT-PCR) analysis

Total RNA was extracted from metformin treated and untreated cell lines using Trizol (Invitrogen) following the manufacturer’s protocol. To study the expression of H2A.Z.1 and H2A.Z.2, 2 μg RNA was reverse-transcribed using High-Capacity cDNA Reverse Transcription Kit (Applied Biosystems). The cDNA was diluted 20-fold for analysis with the specific primer sets (Table 1). The transcript levels were analyzed by SYBR Green incorporation using a Stratagene MX3005P QPCR system and MXPro software. Each 10 μL PCR reaction mixture consisted of 2 μL of diluted cDNA, 2.5 pmol of each primer and 5 μL of Platinum SYBR Green qPCR SuperMix (Invitrogen). Thermocycling conditions for all primer sets were: 9 min 95° C followed by 40 cycles of 15 s 95°C, 30 s 60°C and 45 s 72°C. Relative mRNA levels were calculated using the comparative Ct method (ΔΔCt), considering PCR efficiency. Fold change is equal to 10 ΔΔCt/m, where m is the average slope of the calibration curves for the gene of interest and the endogenous control (GAPDH and PP1). The expression of H2A.Z.1 and H2A.Z.2 transcripts for each sample is represented as fold change relative to this standard.

### Chromatin immunoprecipitation assay (ChIP)

Samples for ChIP were prepared by fixing the cells with 1% formaldehyde for 5 minutes at room temperature; excess formaldehyde was quenched with 125 mM glycine. Cells were washed twice with iced cold Phosphate Buffer Saline (PBS) (pH 7.4). Following washes, cells were pelleted at 600 X g for 5 min at 4°C and cell pellet was resuspended in cell lysis buffer [10 mM HEPES-KOH (pH 7.9), 1.5 mM MgCl_2_, 10 mM KCl, 0.5 mM DDT, 0.1% NP-40 and complete protease inhibitor (Roche Molecular Biochemicals) was added at each step at dilution of 1:100)] and incubated on ice for 20 minutes. The nuclei pellet obtained post centrifugation was resuspended in 6 volumes of 1X radioimmunoprecipitation assay (RIPA) buffer [50mM Tris-HCl (pH 8.0), 150 mM NaCl, 0.1% SDS, 0.5% sodium deoxycholate, 1% Triton X-100, and protease inhibitors]. The resulting nuclei were sonicated to shear the DNA to fragment lengths of 200 to 500 base pairs using bath sonication (Diagenode bioruptor) for 20 minutes with 15 seconds on/off cycles. 5 % of the diluted lysate was reserved as the input and the remainder was pre-cleared with 25 μL of protein G Beads (Dynabeads, Invitrogen Life Technologies). Chromatin was immunoprecipitated (IP) overnight at 4°C with pre-cleared chromatin samples in 1X RIPA buffer containing 0.01% SDS concentration against 3 μg IgG (CST), 3 μg H2A.Z antibody, 2 μg EZH2 and 1μg H3 antibodies, respectively. 25 μL of magnetic beads were added to each IP sample and incubated 4 hours for bead binding at 4°C. Bead: antibody complexes were captured by placing the tubes on a horizontal bar magnet followed by washing of beads twice with low salt buffer [Tris-HCl 50 mM (pH 8.0), NaCl 150 mM, SDS 0.1%, NP-40 1%, EDTA 1 mM, Sodium deoxycholate 0.5%], high salt buffer [Tris-HCl 50 mM (pH 8.0), NaCl 500 mM, SDS 0.1%, NP-40 1%, EDTA 1mM, Sodium deoxycholate 0.5%], LiCl buffer [Tris-HCl 50 mM (pH 8.0), LiCl 250 mM, SDS 0.1%, NP-40 1%, EDTA 1 mM, Sodium deoxycholate 0.5%] and TE buffer [Tris-HCl 10 mM (pH 8.0), EDTA 0.25 mM]. The samples were reverse crosslinked in elution buffer (100 mM NaHCO_3_ and 1% SDS) at 65°C overnight and then incubated with Proteinase K (10 μg/ml final) for 1 hour at 42°C. The DNA was purified using a DNA purification kit (Qiagen) and subjected to qPCR using the primer sequences mentioned in Table 1. Enrichment of DNA fragments after ChIP were normalized to input DNA and to the values obtained with normal rabbit IgG. In addition, immunoprecipitation using H2A.Z was further normalized against H3.

### si-RNA mediated knock down

LNCaP cells were transfected with predesigned small interfering RNA (siRNA) reagents against H2A.Z.1 (*H2AFZ*) and non-targeted (negative) siRNA (Sigma) according to the manufacturer’s protocol. Briefly, 1 × 10^5^ cells/well were plated in six-well plates and grown in RPMI 1640 media supplemented with 10% BGS, 1% penicillin/streptomycin and 1X Gibco GlutaMAX. Cells were transfected with the siRNA duplexes at a final concentration of 30 nM using Lipofectamine reagent (Invitrogen) according to the manufacturer's instructions. After transfection, cells were grown for 24 hours and then treated with the 1mM metformin for an additional 24 hours with no change in media. Cells were harvested and processed for RNA and protein extractions to verify knockdown efficiency.

### Graphic representation and statistical analysis

Graphic (bar plot) representation of the western blot and RT-PCR analyses was carried out using GraphPad Prism version 5. Data is presented as mean +/- standard error. Student’s t-test was used to analyse the significance of difference. All tests were two sided and p < 0.05 was considered significant.
